# Large-scale application of named entity recognition to biomedicine and epidemiology

**DOI:** 10.1371/journal.pdig.0000152

**Published:** 2022-12-07

**Authors:** Shaina Raza, Deepak John Reji, Femi Shajan, Syed Raza Bashir

**Affiliations:** 1 Dalla Lana School of Public Health, University of Toronto, Toronto, Ontario, Canada; 2 Environmental Resources Management, Bangalore, India; 3 Toronto Metropolitan University, Toronto, Ontario, Canada; Harvard University T H Chan School of Public Health, UNITED STATES

## Abstract

**Background:**

Despite significant advancements in biomedical named entity recognition methods, the clinical application of these systems continues to face many challenges: (1) most of the methods are trained on a limited set of clinical entities; (2) these methods are heavily reliant on a large amount of data for both pre-training and prediction, making their use in production impractical; (3) they do not consider non-clinical entities, which are also related to patient’s health, such as social, economic or demographic factors.

**Methods:**

In this paper, we develop Bio-Epidemiology-NER (https://pypi.org/project/Bio-Epidemiology-NER/) an open-source Python package for detecting biomedical named entities from the text. This approach is based on a Transformer-based system and trained on a dataset that is annotated with many named entities (medical, clinical, biomedical, and epidemiological). This approach improves on previous efforts in three ways: (1) it recognizes many clinical entity types, such as medical risk factors, vital signs, drugs, and biological functions; (2) it is easily configurable, reusable, and can scale up for training and inference; (3) it also considers non-clinical factors (age and gender, race and social history and so) that influence health outcomes. At a high level, it consists of the phases: pre-processing, data parsing, named entity recognition, and named entity enhancement.

**Results:**

Experimental results show that our pipeline outperforms other methods on three benchmark datasets with macro-and micro average F1 scores around 90 percent and above.

**Conclusion:**

This package is made publicly available for researchers, doctors, clinicians, and anyone to extract biomedical named entities from unstructured biomedical texts.

## Author summary

This paper introduces and presents a python package https://pypi.org/project/Bio-Epidemiology-NER/ that can extract named entities from biomedical texts. Different from previous works, this package extracts not only clinical entities, such as disease, signs, and symptoms, but also demographics of the patients from the texts. This package can be used with minor code requirements and by epidemiologists, doctors, practitioners, or others in the field to see the named entities from texts. The knowledge gained from the named entities helps the end users see the statistics or spread of infectious disease in the least time and while parsing many free texts.

## 1 Introduction

**Fig 1:**
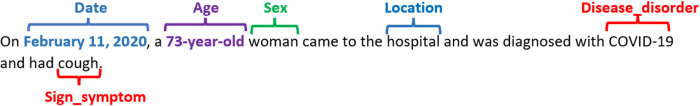
Example of the medical records NER result.

Named entity recognition (NER) [1], a subtask of Natural Language Processing (NLP), seeks to identify and classify named entities (such as person, place, and event) in the unstructured text into pre-defined categories. In the biomedical domain, a fundamental task of NLP is the recognition of named entities, such as genes, diseases, species, chemicals, medical codes, drug names, and so [2]. NER can extract meaningful information from biomedical and clinical texts that can be used for many purposes, such as to study the statistical significance of certain entities (diseases, conditions), events, classification, or relation extraction tasks [3]. The recognition of clinical information through NER on a typical medical record is shown in Fig 1.

As illustrated in Fig 1, it is possible to extract medical information from a typical medical record, such as disease disorder or signs/symptoms, as well as personal demographics (age, sex, location). The state-of-the-art work [3–5] in biomedical NER mainly focuses on limited named entities (disease, chemicals, genes, etc.,). However, many biomedical entities must be considered, particularly those related to clinical diagnoses, such as disease, symptoms, medical concepts, risk factors, and vital signs; epidemiological entities, such as infectious diseases or patient demographics. This research is motivated by the need for an efficient and comprehensive biomedical NER that can automatically extract many entity types (clinical, epidemiological, demographics) from free texts (medical records, electronic health records, published literature) [6] in multiple formats (text, PDFs, rich text). This research aims to facilitate medical practitioners, clinicians, nurses, and doctors in the fast retrieval of information with high accuracy and efficiency.

In the state-of-the-art, the NER models are categorized into three main methods: early rule-based and dictionary-based methods [7], statistical machine-learning-based methods [6], and deep-learning-based methods [2,8,9] in recent years. Although rule-based methods have demonstrated high accuracy, they require many rules written by subject-matter experts, which is a limitation. Statistical machine learning methods also require an annotated corpus, which is not always feasible for large training tasks and limited resources (time and annotators). Deep learning methods have demonstrated powerful generalization ability in recent years and have become the mainstream method for solving NER tasks [10]. The development of hardware capabilities, the emergence of distributed word representations, and the availability of large training corpora have contributed significantly to the success of deep neural network-based methods [11,12].

One of the most effective deep-neural network-based models is Bidirectional Encoder Representations from Transformers (BERT) [13], which is a multi-layer Transformer [12] model with self-attention [14]. The original BERT model was trained on vast quantities of data for more than 104 languages, making its representations applicable to many smaller and similar downstream tasks, such as classification, NER, and relation extraction. Research shows that the distillation of a large language model can yield almost the same results as the original model, but we benefit from better efficiency and ease of use for production [15]. In this research, we employ DistilBERT [16], a simplified version of the BERT with fewer parameters, faster training, and better performance for the task of biomedical NER. Our contribution to this research is three-fold:

1. We developed a python package BioEN, short for **Bio**-**E**pidemiology-**N**er (https://pypi.org/project/Bio-Epidemiology-NER/), which can recognize accurate biomedical named entity annotations from the free text data (medical records, clinical notes, case reports, scientific publications). This package can parse text data in various input formats, including text files, tabular data, and PDF files. To facilitate analysis for end users, the model outputs named entities in both data frames and annotated PDF formats (if the input is a PDF file). The novelty of this work is in the subtle integration of many NLP models that are stacked into a package for ease-of-use for the medical community and the researchers.
2. We make this package publicly available for distribution as software tools via PyPI and pip, making it easy for developers, researchers, and anyone with minimal programming knowledge to download and install it for simple experiments and large, professional systems.
3. We provide many biomedical entity types, such as diseases, risk factors, adverse events, and patient demographics, which are both extensive and more informative in comparison to previous works in this line of research.

Experimental results on several benchmark datasets showed the superiority of our approach compared to the state-of-the-art methods.

The rest of the paper is organized as follows: section 2 is the related work, section 3 is the methodology, section 4 is the experimental setup, section 5 is the results and analysis, section 6 is the discussion, and section 7 is the conclusion.

## 2 Related work

Named entity recognition (NER) is the task of identifying a named entity (a real-world object or concept) in unstructured text and then classifying the entity into a standard category [1]. These methods involve two tasks: (1) identification of entities (e.g., persons, organizations, locations, etc.) in text, and (2) classification of these entities into a set of pre-defined categories, such as person names, organizations (companies, government organizations, committees, etc.), locations (cities, countries, towns), date and time [1]. Traditional NER methods only consider specific entities. However, there can be more entities, depending on the domain used. For example, the field of biomedicine covers entities such as genes, diseases, chemicals, and proteins [2].

In recent years, there has been a dramatic increase in biomedical data [17]. Due to the COVID-19 surge, there has been a massive increase in biomedical data in the last two years. Such enormous data is challenging to process, significantly when the urgency of time and the number of patients is increasing exponentially. [18]. To perform biomedical mining, it is essential to accommodate a prior process of biomedical NER. Biomedical NER is the task of identifying entities in the biomedical domain, such as chemical compounds, genes, proteins, viruses, disorders, drugs, adverse effects, metabolites, diseases, tissues, DNAs and RNAs, organs, toxins, food, or so [3,10]. Most research [1,3,19] in biomedical NER focus on general approaches to named entities that are not specific to the biomedical field. On the other hand, some works [20–22] focus solely on biomedical and chemical NER; however, they don’t cover many clinical entities. In this research, we plan to cover many named entities, that are both clinical, biomedical and epidemiological.

Word embedding is a valuable technique that uses a large amount of unlabeled data to learn the latent syntactic and semantic information of words/tokens and map these words/tokens into dense low-dimensional vectors. In the past few years, many word embedding methods, such Word2Vec [23] and GloVe [24] are proposed. Unlike traditional word embeddings such as Word2Vec and GloVe, the embedding assigned to the word/token by the language mode, such as ELMo [25] and BERT [13] depends on the context, which means the same word/token could have different representations in different contexts. BERT employs Transformer [14] to pre-train word representations by jointly conditioning on both the left and right context in all layers. Because of the great success of BERT, it has gradually become a mainstream method using a large corpus to pre-train BERT and fine-tunes it on the target dataset. The BERT is also a widely used model for many downstream tasks, such as NER and relation extractions.

Some biomedical works consider BERT for the biomedical NER tasks [5,26] and have shown outstanding performance. In this work, we also use the BERT model for NER task but the distilled version of the BERT, DistilBERT [16]. The DistilBERT retains only half of the actual BERT model’s layers and parameters. The distilled versions also balance the computational complexity and the accuracy of the model, which is the motivation for our model building.

## 3 Materials and methods

### 3.1 Problem definition

Given an input sentence *X* = {*x*_1_, *x*_2_,….,*x*_*N*_}, where *x*_*i*_ is the *i*^th^ word (token), and *N* represents the length of the sentence. The goal of this study is to classify each token in *X* and assign it to a corresponding label *y*∈*Y*, where *Y* is a pre-defined list of all possible label types (e.g., disease, symptoms, drugs etc.).

**Fig 2:**
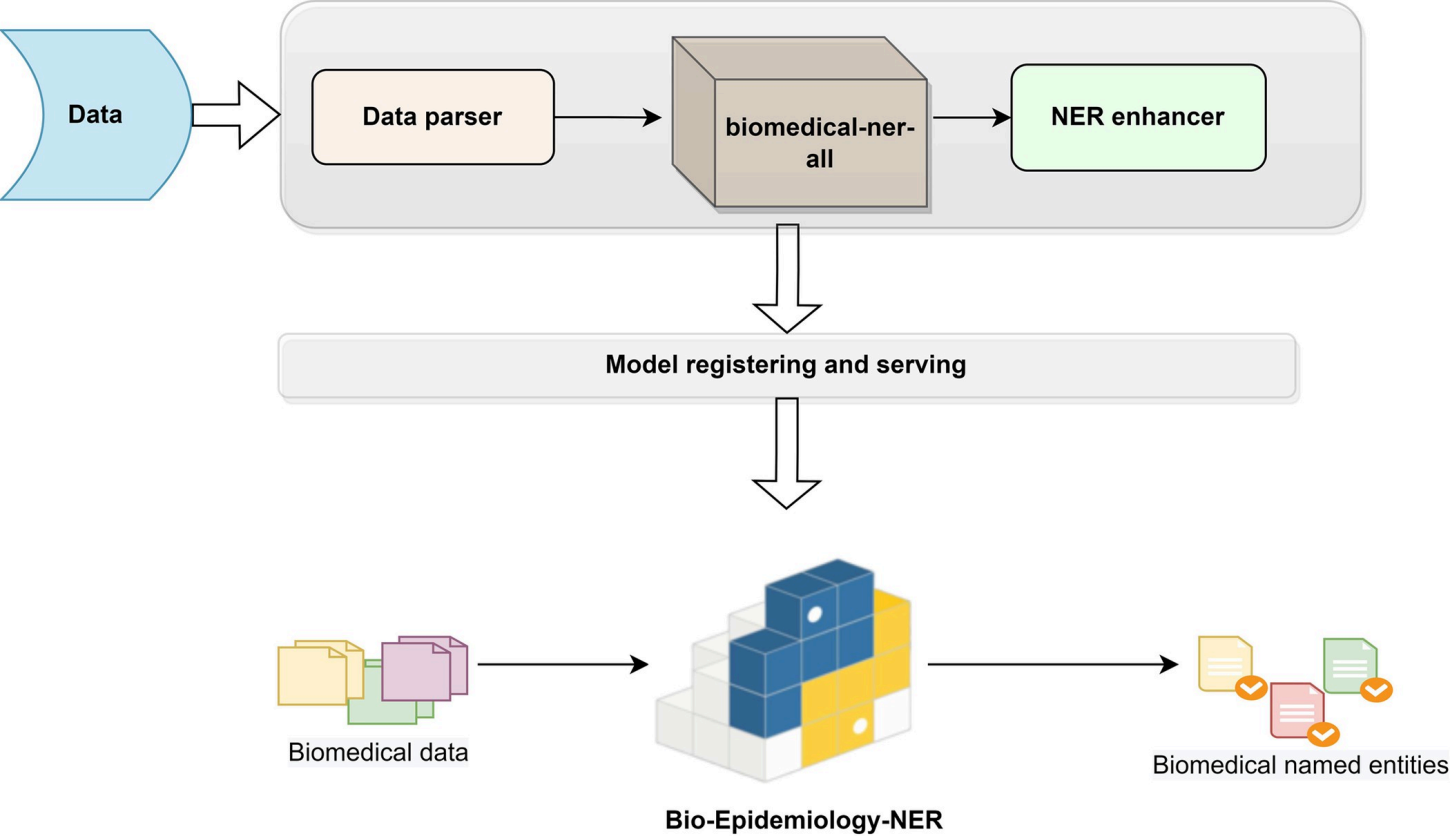
BioEN ((Bio-Epidemiology-NER) Development Architecture.

Next, we present the workflow of BioEN development architecture in Fig 2.

### 3.2 Overall architecture

We have proposed and developed the BioEN package with a 3-phased approach in this study, as shown in Fig 2.

The detail of each phase is given below:

First, we get the data, which is free text, and we feed the biomedical data to BioEN. In the first phase, the solution phase (starting from the top) of the architecture, we build a fine-tuned Transformer model (we name it the biomedical-ner-all model) with a data parser and a NER enhancer. The second phase is the model registering and serving to prepare the models and related components for packaging. The third phase is the production phase, where we make our python package ready and deploy it for real-time use. We explain the phases of BioEN architecture below:

#### 3.2.1 Solution phase

The first phase is the solution phase, where we have the data parser component, model processing, and final output generation. We name this phase the ‘solution’ phase since we provide the leading solutions to biomedical NER tasks here.

*Data ingestion*. The solution phase starts it working with the data ingestion. We can feed free (unstructured) texts in any format (text files, rich texts, pdf files) to the data ingestion step. In this study, we feed the biomedical data (details in Section 4.1). However, we design this architecture with reusability in mind so that the same architecture can be used with any other domain-specific data (e.g., life sciences, news feeds, entertainment).

*Data parser*. The free texts after data ingestion go into the *data parser* module. Data parsing is converting data strings from one format to another that is readable by the following components in this phase. The data parser module can extract textual data from any format, including PDF documents. Such a multipurpose data parser assumes that most biomedicine articles are available in PDF file formats.

*Transformer-based model*, *biomedical-ner-all*. The pre-processed texts from the data parser go into the next module, a Transformer-based module. We name this module the *biomedical-ner-all* model, and we make this module available at https://huggingface.co/d4data/biomedical-ner-all. The biomedical-ner-all model can identify the biomedical-named entities from the texts. We have fine-tuned this model on biomedicine data that covers a large number of clinical, epidemiological, and non-clinical (demographics) named entities. More details about biomedical-ner-all in Section 3.3.

*NER enhancer*. The output of the last module (i.e., Transformer-based model) is in the IOB (Inside-Outside-Before) format, which has become a prototypical standard format for tagging tokens in the NER tasks [27]. The NER enhancer module is built on top of the biomedical-ner-all model and converts the IOB representation to a user-friendly format by associating chunks (tokens of recognized named entities) with their respective labels and enhancing the NER predictions. We filter out the NER chunks with no associated entity (tagged ‘O’). The output of the ner enhancer module is the named entities that are easily readable and tagged. For example, we tag the exact token place for a disease mention, a symptom, or any other named entities as output representations.

#### 3.2.2 Model registering and serving

The second phase in the BioEN development architecture is the model registering and serving. In this phase, we bundle our ML models (in the first phase) into a package for local real-time inference and batch inference. The idea is to support the real-time data processing and model serving workflow. We register the model and its components in a standard format for packaging (as in PyPi and pip) that can be used in various downstream tools—for example, batch inference or real-time serving through a REST API. By the end of this phase, we will have a package and a hosted model that can be called using a generalized code snippet, and we can load and run the model for the biomedical NER task.

#### 3.2.3 Biomedical-epidemiology-NER

The third phase in BioEN development architecture is the production phase. In this phase, we handle the versioning of our package and upload the final version to PyPI for distribution. The idea is to make this package available open-source so that other developers, biostatisticians, epidemiologists, or researchers can easily download and install it for simple experiments or as part of large, professional systems. The package is available under MIT license with this link https://pypi.org/project/Bio-Epidemiology-NER/ and can be installed in any python environment by the following simple python command**: pip install Bio-Epidemiology-NER**

**Fig 3:**
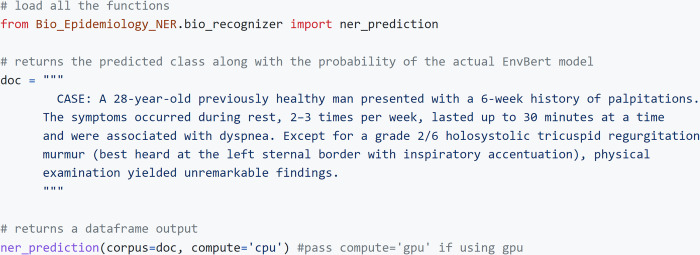
Illustration of package use on a case report.

Below, we show the use of this package, which is quite simple, in Fig 3.

The results are saved either in dataframes as CSV files with each chunk and named entity as output, or in PDF files with entities marked and shown. The tutorial and further details for use are given in the package website.

#### 3.2.4 Biomedical named entities

The package output is biomedical named entities that are provided to the user in two formats: 1) data frames in CSV format with each word/ chunk and the identified named entity, and 2) pdf file with named entities highlighted and annotated in the texts.

In the state-of-the-art biomedicine works, there are usually a few named entities, such as diseases, genes, proteins, chemicals or species. However, we provide many clinical, non-clinical (demographics), and epidemiological named entities related to infectious diseases and the events, which we claim to be our unique contribution. We list the named entities that we are using in this work in Table 1:

**Table 1:** Named entities used in this work.

Activity	Diagnostic_procedure	Shape
Administration	Disease_disorder	Frequency
Age	Distance	Subject
Personal_background	Sign_symptom	Texture
Family_history	Medication	Therapeutic_procedure
Height	Outcome	Time
Sex	Severity	Volume
Color	Lab_value	Weight
Date	Mass	Dosage
Non-biological_location	History	Duration
Detailed_description	Co-reference	Biological_attribute
Occupation	Qualitative_concept	Biological_structure
Personal- Biological_structure	Quantitative_concept	Clinical_event
	Area	Other_entity

Next, we explain our methodology and discuss our Transformer-based model ***biomedical-ner-all*** in detail.

### 3.3 Model details

**Fig 4:**
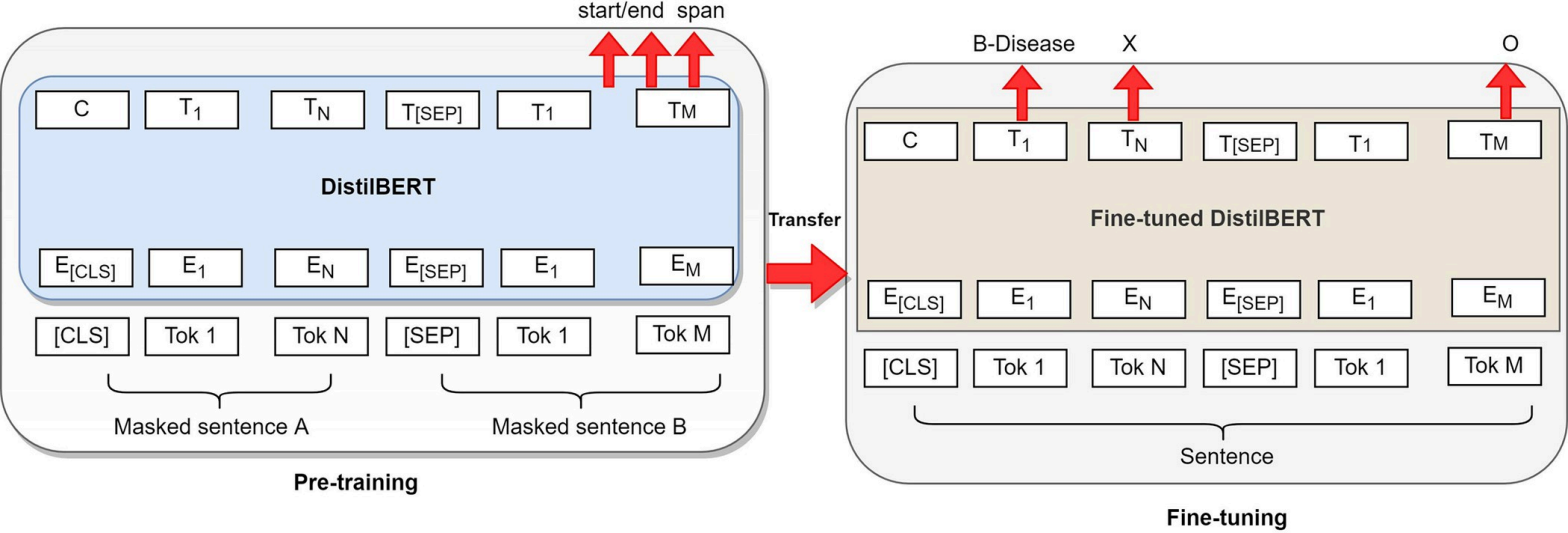
Model adaption from the teacher model to the student model.

BERT and its refined version, DistilBERT [16], are Transformer-based models with self-attention blocks. These models are pre-trained on unlabeled raw texts and can be used for various tasks, including question answering, sentence-pair classification, and sequence tagging tasks [28]. The idea is to have a general architecture that applies to many problems and a pre-trained model that reduces the need for labeled data. This study fine-tunes the pre-trained DistilBERT model to learn a more accurate representation of target domain entities (biomedicine entities). We prefer the fine-tuning over the pre-training task that is more expensive than fine-tuning. In particular, we change the last layer of the DistilBERT model for our specific biomedical task and fine-tune it on our dataset. We show the model adaption from a teacher model (pre-trained DistilBERT) to our student model (fine-tuned DistilBERT) in Fig 4.

As shown in Fig 4, we fine-tine the pre-trained DistilBERT model’s weights for the initialization of the NER task, and we adjust the input and output to our biomedical task. We release the model weights of our fine-tuned model here [29]. The model weights are also packaged in our python package [30].

In the fine-tuning stage, we replace the entity tagging head of DistilBERT with a randomly initialized new head that covers all the entity categories of the target domain. We fine-tune the model using the target domain (biomedicine) training data. The model is optimized with Adam [31] with a batch size of 16 and a learning rate of 2e-5. We train the model for 40 epochs and evaluate the model on the development set using entity-level F1-score on sub-word tokens. In addition to early stopping, the model is regularized with dropout within each transformer block and weight decay. The dropout is set to 0.1, and the weight decay is set to be 0.01 in the experiments. The best-performing model weights is used as the final prediction model.

**Fig 5:**
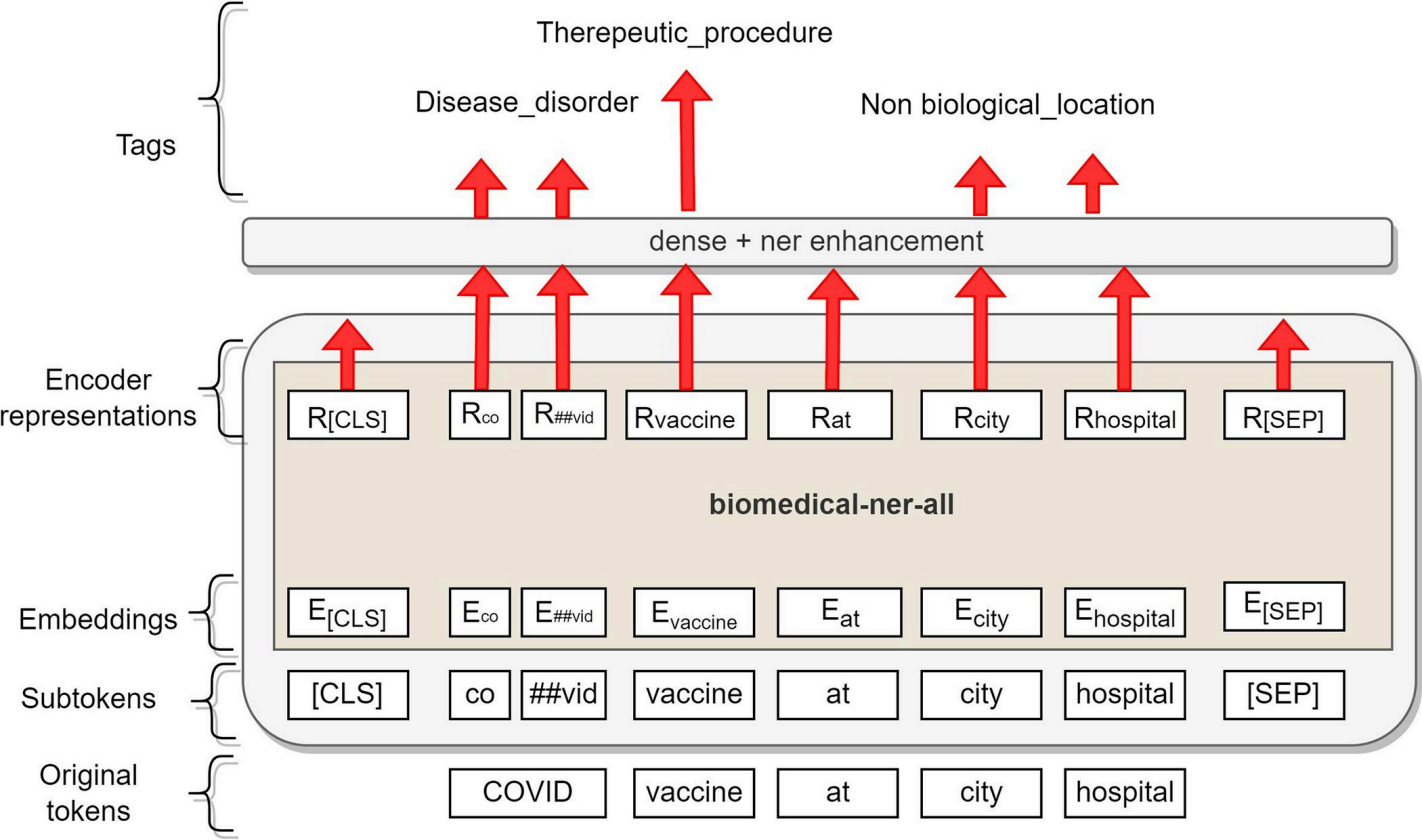
biomedical-ner-all model.

As the input for the BERT model, we use the CoNLL-2003 [32] formatted data, split into sentences and tokenized on the word level. Sentences more prolonged than the limit (512 sequences) are split into separate input sequences for the network. When converting the predictions back to the word-level CoNLL format, we assign the predicted entity label of the first sub-word unit for the entire token. Our fine-tuned model, which we name as ‘biomedical-ner-all’ released with our package also, is shown in Fig 5.

As shown in Fig 5, we give the input, sequences of texts to the model. The input is tokenized, and its input representation is constructed for each given token by summing the corresponding token, segment, and position embeddings. The embedding of words goes as input to the dense layer, where we perform the enhancements to convert the IOB format of CONLL-2003 to a user-friendly format. The output is the set of entities that are user-friendly and easily readable. Along with extracting each entity, our model also shows a confidence score. A confidence score is simply a decimal number between 0 and 1 and indicates how confident the system is with its prediction. We consider only the entities with a prediction confidence score of more than 0.4 in the final output.

### 3.4 Data

In this work, we use the publicly available data MACROBBAT 2020 dataset [33]. The dataset details are available in the original paper [34], where the authors define the acronym ACROBBAT as ‘Annotation for Case Reports using Open Biomedical Annotation Terms’. The dataset consists of 200 source documents in plain text and 200 annotation documents, each annotation document with plain text. Each document is named using PubMed document identifier, for example, 18258107.txt and 18258107.ann. The authors extracted the document’s text from the PubMed article but only included the clinical case report information, which is also relevant to our application because it contains patients’ data.

As mentioned in the dataset paper [34], the documents were manually annotated by researchers with prior experience reading biomedical and clinical language. Following completion, the annotations were checked for format and type consistency. We fine-tune our Transformer-based model (shown in Fig 5) with the MACROBBAT data, which covers a wide range of named entities (clinical, demographics, epidemiological and event-based). We also update the package with additional training on a portion of COVID-19 data to accurately detect the mentions of the latest Coronavirus and/or COVID-19 named entities. The details of the dataset used in this study to train our model are given in Table 2:

**Table 2:** Dataset details.

No. of documents	200 PubMed documents
**No. of sentences**	3,652
**No. of annotations**	59,164
**Main Entity types**	Clinical events, Diagnostic, procedure, disease disorder, medication, sign symptoms, time expressions, demographics, patient history, temporal and causal relations, co-references.
**Distinct entity types**	42 (given in Table 1)

Besides MACROBBAT, we also evaluate our approach against the following benchmark datasets:

- **NCBI-Disease** [35]: it is the dataset introduced for disease NER and normalization. It has been widely used for a lot of applications. It consists of 793 PubMed abstracts with disease mentions.
- **I2b2-2012** [36]: it consists of clinical (problems, tests, treatments, clinical departments), occurrences (admission, discharge and evidence) mentions using 310 discharge summaries.

We also collected random case reports (around 500) from the LitCOVID [37] data source from 2020–2021 to see the effectiveness of our approach on the biomedical and COVID-19 patients’ data.

### 3.5 Benchmarking methods and evaluation

**Benchmarking**: To assess the utility and challenges of our approach, we evaluate the performance of our NER approach against the following state-of-the-art models:

- **BiLSTM-CNN-Char** [38], a hybrid Bidirectional Long Short-Term Memory (LSTM) and Convolutional Neural Network (CNN) architecture that learns both character and word-level features for the NER task.
- **SciBERT** (Base), a pre-trained language model based on BERT pre-trained on a large multi-domain corpus of scientific publications to improve performance on downstream scientific NLP tasks.
- **BlueBERT** [39], BERT-based uncased model pretrained on PubMed abstracts and MIMIC-III.
- **ClinicalBERT** [40], BERT-Base-cased model trained on MIMIC notes.
- **BioBERT** [5], a pre-trained biomedical language representation model for biomedical text mining. We use the BioBERT-Base v1.2 (+ PubMed 1M).

These models have obtained state-of-the-art performance in their works, respectively.

**Evaluation metrics**: Following the standard practice [2,32,41] to evaluate NER tasks, we use the typical evaluation method, i.e., precision, recall and F1-score at a token level.

- Precision: percentage of named entities found by the learning system that are correct.
- Recall: percentage of named entities present in the data found by the system. A named entity is correct only if it is an exact match of the corresponding entity in the data file.
- F1-score: harmonic mean of precision and recall.

**Configurations and Hyperparameters:** We use PyTorch for the model implementation. We run our experiments on GPU: 1 x GeForce RTX 3060 with 16.0 GB RAM to integrate the components of our package. We use Grid search to get the optimal values for the hyperparameters and early stopping to overcome possible overfitting. We specify the following hyperparameters as shown in Table 3.

**Table 3:** Hyperparameters used.

Hyperparameter	Optimal value used
Learning rate	2E-5
Batch size	16
Epochs	40
Optimizer	Adam
Dropout rate	0.5
Optimizer	Adam
Hidden Size	768
Embedding Size	128
Max Seq Length	512
Warmup Steps	3000
Weight decay	0.01
dropout	0.1

We have divided the dataset into training, validation, and test sets, with a 70:15:15 ratio for all experiments.

## 4 Results

In this section, we present the results and analysis.

### 4.1 Overall performance evaluation

We present the results of our model and the baseline models on all the datasets in Table 4.

**Table 4:** Overall performance.

Method	Metric	MACCROBAT	NCBI-Disease	I2b2-2012
BiLSTM-CNN-Char	Precision	84.43	85.24	79.35
	Recall	83.97	83.31	78.11
	F1-score	84.20	84.26	78.73
SciBERT	Precision	78.10	76.88	77.01
	Recall	72.18	74.10	75.18
	F1-score	75.02	75.46	76.08
BlueBERT	Precision	84.04	83.37	81.10
	Recall	81.48	81.39	80.88
	F1-score	82.74	82.37	80.99
ClinicalBERT	Precision	81.01	84.08	80.35
	Recall	79.10	80.11	78.69
	F1-score	80.04	82.05	79.51
BioBERT v1.2	Precision	*86*.*72*	*85*.*80*	*88*.*00*
	Recall	*88*.*31*	*84*.*29*	*86*.*10*
	F1-score	*87*.*51*	*85*.*04*	*87*.*04*
**BioEN (our approach)**	Precision	**92.10**	**91.68**	**90.10**
	Recall	**91.68**	**88.92**	**88.98**
	F1-score	**91.89**	**90.28**	**89.54**

Table 4 shows that our BioEN approach achieves state-of-the-art performance for detecting many biomedical-named entities. This is demonstrated by our model outperforming other methods by performing around 90% F1-score on all datasets. The superiority of our model is credited to its architecture and carefully fine-tuned biomedical-ner-all model. This result also demonstrates that a model that is trained on domain-specific data (e.g., biomedical data) can be applied to a wide variety of domain-specific terminologies. For example, we train this package BioEN (including its components) on biomedical named entities, and we can detect a large variety of clinical, event-based, and epidemiological named entities to study infectious diseases and population groups. Our model also considers the causal relations and co-references provided by the MACCROBAT dataset on which it is primarily trained. Thus, this package can incorporate a diverse vocabulary and phenomena described in clinical documents without requiring direct connections to curated concepts (e.g., MeSH, UMLS knowledge bases).

We also see that BERT-based methods (BioBERT, BlueBERT, and ClinicalBERT) that have been pre-trained on PubMed and/or MIMIC-III clinical notes perform well. This is most likely because these methods have been well-trained for extracting richer features, resulting in better performance. BioBERT outperforms other BlueBERT, ClinicalBERT, and SciBERT, which is probably because BioBERT is pre-trained on more biomedical and clinical data and can better infer the patterns from the test data. We also find that BlueBERT performance is quite good, above 80%, which shows models pre-trained on domain-specific literature (e.g., biomedicine) perform well in the respective downstream tasks.

As demonstrated in Table 4, BiLSTM-CNN-Char that automatically detects word- and character-level features using a hybrid BiLSTM and CNN architecture yields quite a good result, after BioEN (ours) and BioBERT methods. BiLSTM-CNN-Char model achieves an F1- score performance of approximately 84% on MACCROBAT and NCBI datasets, which are quite high compared to most models. BiLSTM-CNN-Char has also been used as a state-of-the-art and conventional model for many biomedical datasets. However, the latter works show that adding the language models on top of traditional models (e.g., BiLSTM) shows good performance [42,43], which may be attributed to large-scale pre-training tasks on the top.

SciBERT is trained on papers from the semanticscholar.org corpus; while the knowledge gained is significant, the model’s performance in our work is somewhat compromised. We anticipate this comparatively lower performance may result from the limited biomedical literature used to train the model, and the named entity types of the actual model differ in some way from what is expected by our application.

Although we fine-tune each baseline method to its optimal hyperparameter settings, we anticipate that the relatively low scores of these baselines on the MACCROBAT dataset, which is a primary dataset for our model training, can be because of a few factors, for example, lack of complete training, and unavailability of the training/test set splits utilized in previous studies.

### 4.2 Effectiveness of the BioEN on case reports

We give a snippet from a COVID-19-related case report [44] to BioEN (our method) and show the confidence score for the predicted entities. Due to brevity reasons, we show the results only on one sentence from the case report in Table 5.

**Table 5:** The confidence score of the model on different named entities by BioEN.

Entity group	Value	Score
Sex	man	0.999528
History	40s	0.867149
Clinical_event	admitted	0.999799
Location	local hospital	0.997007
Date	6 days after	0.991963
Disease_disorder	COVID-19	1.000000
Diagnostic_procedure	general condition	0.999905
Therapeutic_procedure	treated	0.999675
Location	intensive care unit	0.999643
Sign_symptom	cough	0.999682
Sign_symptom	experienced dyspnoea recurred and rapidly increased	0.999941
Sign_symptom	dyspnoea	0.999938
Sign_symptom	recurred	0.992294
Diagnostic_procedure	CT	0.999842
Biological_structure	pulmonary	0.999952
Diagnostic_procedure	angiogram	0.999405
Area	10×18 cm	0.997637
Detailed_description	cavitary	0.999947
Detailed_description	pulmonary cavitary	0.999942
Detailed_description	air-fluid level	0.999005
Disease_disorder	atelectasis	0.997569
Detailed_description	one-way valve mechanism	0.847769
Co-reference	developed pneumatocele	0.863188
Co-reference	one-way pneumatocele	0.792779
Biological_structure	bronchial endobronchial segments	0.915637
Biological_structure	right lower lobe	0.999896
Biological_structure	endobronchial	0.999509
Date	4 weeks after	0.999853
Date	Six months after	0.999874
Biological_structure	lung	0.980932

As can be seen, our model is able to predict the named entities with quite accuracy, which two experts in the biomedicine field also validate.

### 4.3 Case study

We show our model’s most frequent top-10 named entity types predicted by parsing 100 random case reports from PubMed in Table 6. These case reports are selected from the timeline 2020–2021 and are related to COVID-19. These results show the predictions from our model.

**Table 6:** Most frequent named entities from 100 covid-19 case reports.

Disease disorder	Sign symptom	Therapeutic procedure	Medication
covid-19	cough	arterial	aspirin
homocystinuria	fever	Endomyocardial	homocysteine
influenza	shortness of breath	intubation	alcohol-based
asthma	sore throat	oxygenation	Paxlovid
SARS	headache	splenectomy	Tylenol
gastroenteritis	aches and pains	vaccination	ibuprofen
long-COVID	diarrhoea	venoarterial	clopidogrel
cardiac	chest pain	Myocardial	prasugrel
repiratory	rash	Endovascular	acetaminophen
pneumonia	difficulty breathing	transplantation	antiviral pills

These results in Table 6 can be used to get valuable information regarding the most frequent disorders or symptoms mentioned in the case report or to find the most common findings efficiently. According to these results, the most common symptom is cough and fever, while aspirin is the most common drug ingredient. The most common disease disorder here is COVID-19 since these are COVID-19 case reports.

**Fig 6:**
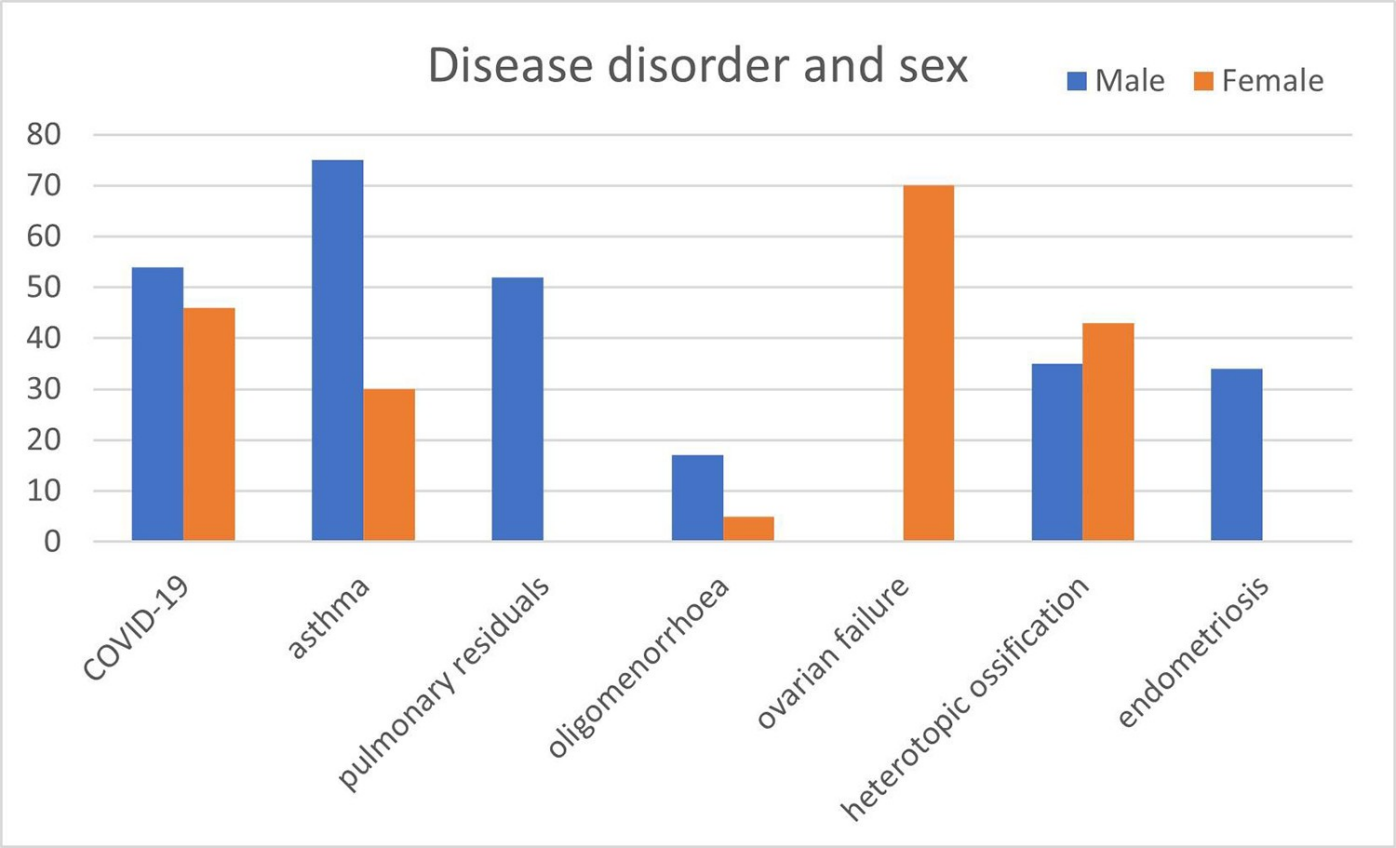
Distribution of disease disorders among male and female groups.

We also show the most common disease disorder in two sexes (male and female), based on 100 case reports data, and the results are shown in Fig 6.

As shown in Fig 6, COVID-19 patients around 54% as male, and the others are females. Ovarian failure, a female disease order, is found in female patients only. For the other diseases, we see asthma mostly in male patients and endometriosis only in male patients.

**Fig 7:**
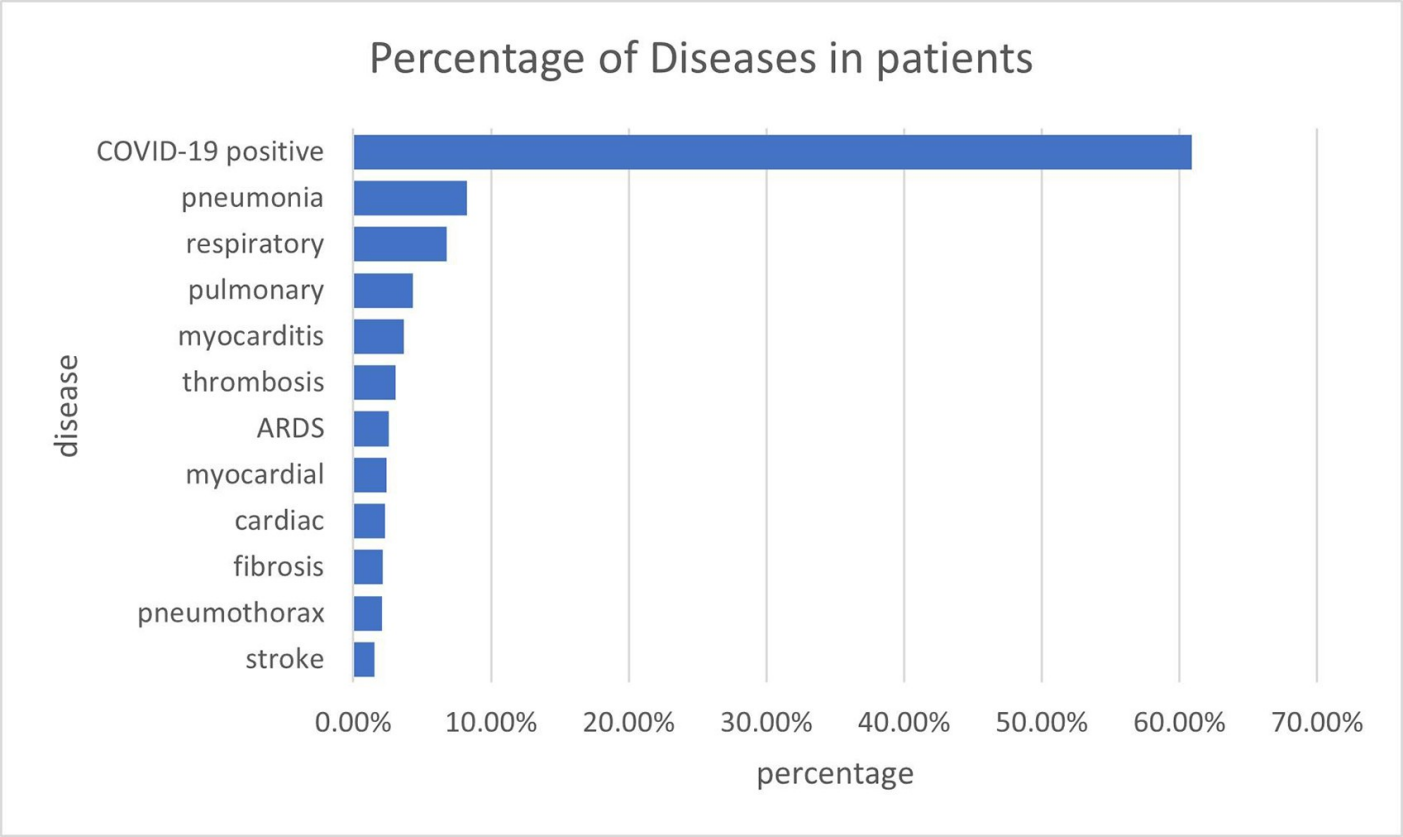
Percentage of most occurring disease disorders in patients.

We also show the percentage of most occurring diseases in the patients in Fig 7, and the most frequent diseases are COVID-19 positive, pneumonia, and respiratory, which are primarily related to COVID-19 disease.

**Fig 8:**
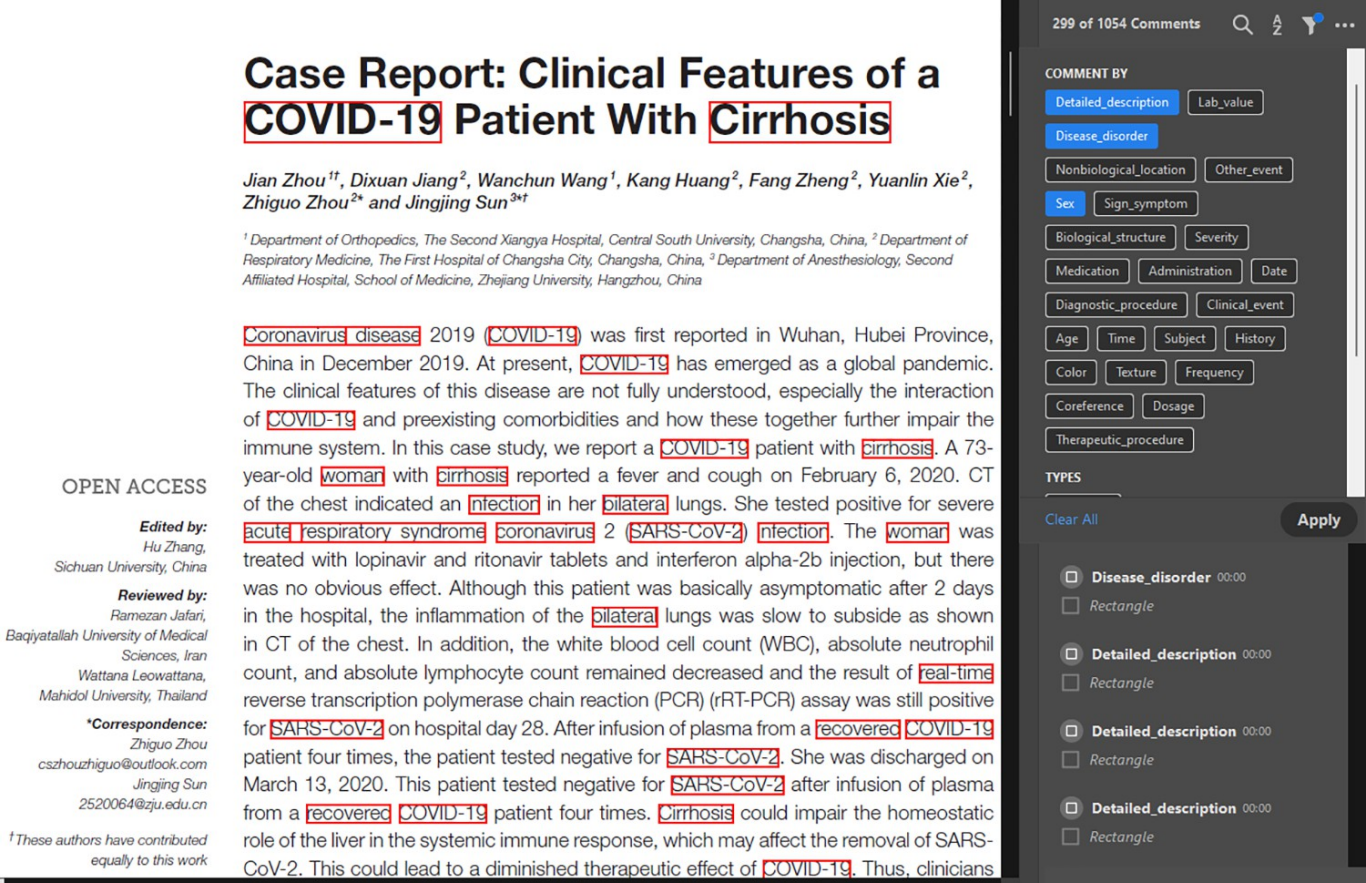
Annotations produced by our model in PDF file.

We show a snippet of one case report in PDF format that is parsed by our BioEN model, and the results are saved and annotated in the same PDF file as shown in Fig 8.

## 5 Discussions

This package’s results and findings can be used in healthcare applications, such as assisting doctors, nurses, and clinical experts in matching symptoms to diagnosis, treatment, and follow-up. This package is simple to install and configurable for real-time use by medical practitioners. The model’s results apply to various applications, for example, disease detection, demographic studies, social determinants of health, semantic relation extraction between concepts in medicine biology, and other related tasks. BioEN also impacts application performance in terms of precision and recall.

Healthcare and health science data face numerous challenges in the “big data” era. With this approach, we attempt to provide automatic methods for text and data mining tools that must be deployed to deal with large, highly heterogeneous data sets. As the field of NLP advances, policymakers will have more opportunities to understand the value of electronic medical records and clinical records, as well as the cost-effectiveness and cost-savings implications of health system planning. This solution allows for the tracking of medical as well as social determinants of health, which can lead to the reduction of health disparities. However, more research is required to understand and assess the dataset better.

*Limitations*: So far, we rely on benchmark data to train the model. However, more data is required to train the model to study many infectious diseases. In the future, we plan to annotate our biomedical data, and we strongly encourage the inclusion of medical professionals in the annotation guideline. We also intend to curate more clinical data; in particular, getting real-time access to EHRs would be helpful. Due to the black-box nature of most deep neural networks, we also plan to handle bias or systematic error in research methods, which may influence disease associations and predictions. We also plan to consider the human evaluation to have predicted entities as being more accurate, informative, and biomedical. We plan to make the model multi-lingual to consider more text from other languages and avoid any bias towards a single language.

## 6 Conclusion

In conclusion, this paper presents BioEN development architecture consisting of several components stacked together. We use an approach to train models for the biomedical named entities by fine-tuning the BERT-based Transformer model. We fine-tune a Transformer based architecture to the task of biomedical NER. We evaluate the performance of our approach in different benchmark datasets, and our method achieves state-of-the-art results compared to the baselines. We demonstrate through extensive experiments that using contextualized word embedding pre-trained on biomedical corpora significantly improves the outcomes in NER tasks.
